# Novel high‐affinity EGFRvIII‐specific chimeric antigen receptor T cells effectively eliminate human glioblastoma

**DOI:** 10.1002/cti2.1317

**Published:** 2021-07-18

**Authors:** Rebecca C Abbott, Daniel J Verdon, Fiona M Gracey, Hannah E Hughes‐Parry, Melinda Iliopoulos, Katherine A Watson, Matthias Mulazzani, Kylie Luong, Colleen D’Arcy, Lucy C Sullivan, Ben R Kiefel, Ryan S Cross, Misty R Jenkins

*Clinical & Translational Immunology* 2021; **10**: e1317.

**Correction to:** *Clin Trans Immunol* 2021; **10**: e1283.

https://doi.org/10.1002/cti2.1283. Published online 9 May 2021

Figure 5 originally published in this article was incorrect, as it contained a duplicated mouse image. The corrected Figure 5 and its caption appear below.

The authors apologise for this error.

**Figure:**
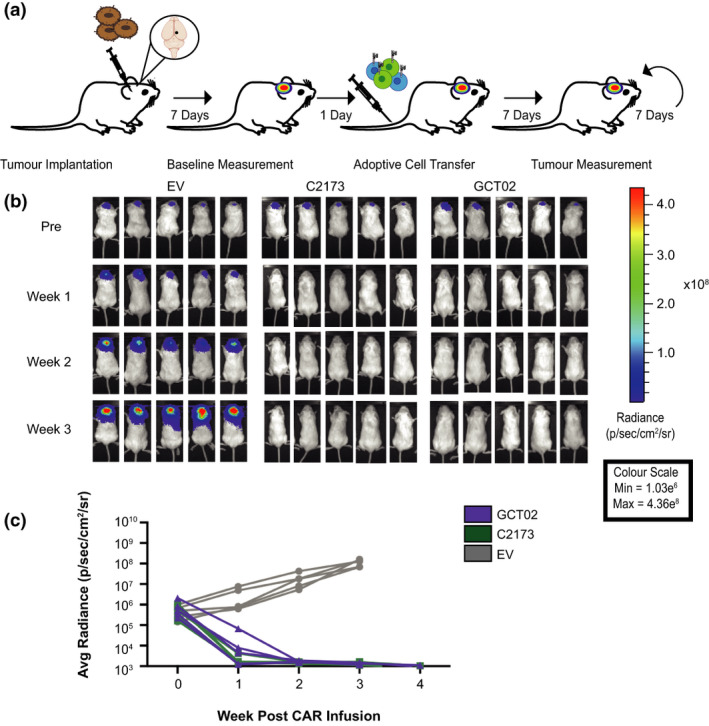
GCT02 CAR T cells effectively induce regression of intracranial tumors. **(a)** Schematic of the experimental protocol to evaluate the *in vivo* function of CAR T cells against EGFRvIII‐expressing intracranial tumors. Mice were intracranially injected with U87‐EGFRvIII GFP‐Luc tumor cells and 7 days later were imaged using bioluminescence. The mice were allocated to treatment groups before delivery of a single intravenous dose of 5 × 10^6^ CD4^+^: 5 × 10^6^ CD8^+^ T cells day 8 post‐activation GCT02 or C2173 CAR T cells. Empty vector T cells (EV) were injected as a negative control. Bioluminescence was examined weekly to monitor tumor size over time. The tumor injection site is indicated by black circle. **(b)** Bioluminescence imaging of U87‐EGFRvIII GFP‐Luc tumor‐bearing NSG mice, treated with either empty vector (EV), C2173 or GCT02 CAR T cells. Individual mice from each treatment group are shown for up to 3 weeks after CAR T cell infusion. Representative of two independent experiments. **(c)** Quantification of tumor growth in the mice in panel **b**. Tumor size was quantitated in radiance (photons/sec/area/sr). Each line represents a single mouse. *n* = 5 mice per group. Data are one representative of two independent biological replicates.

